# Dental Implants in the Third Millennium

**DOI:** 10.1155/2018/9752141

**Published:** 2018-05-02

**Authors:** Luigi Canullo, Eitan Mijiritsky, Silvio Mario Meloni

**Affiliations:** ^1^University of Valencia, Valencia, Spain; ^2^Maxillofacial Surgery, Department of Otolaryngology, Tel-Aviv Sourasky Medical Center, Sackler Faculty of Medicine, Tel-Aviv University, Tel Aviv, Israel; ^3^School of Dentistry, University of Sassari, Sassari, Italy

From Galileo to Bacon, the science has always tried to bring the scientific discipline on the road of “evidence” in face of many possible and arbitrary “opinions.” For instance, the Cochrane Library exists so that healthcare decisions could get better. This organization tried to certify this “evidence” using a methodologic approach. Nevertheless, in oral and maxillofacial surgery, most of systematic reviews founded weak or no evidence that any particular type of intervention is better than any other.

Evidence medicine (EBM) has been defined as “the judicious use of the best current evidence in making decisions about the care of the individual patient” [1]. It was assumed that only randomized trials can generate level I evidence. Particularly, due to its rigor in design and control for different types of bias, randomized double-blind placebo controlled trials have had the largest impact in leading clinicians toward the concept of EBM. However, other methods, such as prospective cohort studies, can also be used for evidence-based decision making.

The rationale behind the EBM is to analyze only the effect of the drug, removing the patients' and operators' variables. To cancel patient variability, sample size should be in the order of thousands. At the same time, the only skill required to the operator is to display a pill. EBM has been applied to surgery over the past decades with increasing numbers of randomized controlled trials (RCTs). Nevertheless, interpretation of its clinical meaning might be problematic in the dental surgical field. In fact, despite a test technique can be compared to a control one in a randomized modality, to ensure that the techniques can be precisely defined and then performed in a standardized manner for each operator remains challenging. The skill of the operator, which remains the most important variable, must not be underestimated. Finally, due to the ethical conflicts intrinsic to the surgical field, sample size is very often small, and despite statistical tricky analyses and strict inclusion/exclusion medical criteria, patient variability cannot be excluded. Very often, in fact, to statistically increase numbers and apparently generalize the meaning of the message, the biology behind the cases is underestimated and the outcomes are so generalized to lose a specific clinical effectiveness.

Although the start of this methodologic approach was really promising, its, so far, extensive and maybe misinterpreted use in the surgical field might represent only a tricky way to publish.

Controversially, in the modern implant dentistry, bio-logic (a composite neologism to express the need to combine knowledge of biology and the use of the logic) could be the key factor to interpret and choose the correct surgical (and not only) techniques to treat a clinical case. At the light of the third millennium clinical demands, interpretation of the evidence in surgical dentistry through Cochrane's guidelines might appear problematic due to the nature of surgery itself. Outcomes of systematic reviews (at least with the actual standards) should be used as approximate indications.

Randomized controlled trial and systematic review will continue to be a safety margin not to go beyond, but the message to spread to the scientific community should be to encourage the production of long-term prospective and also retrospective study conducted in clinical practice. These might provide less high levels but without forgetting the importance of the experience and the operator skill, just focusing on the potential limits and complications of the technique itself.

*Luigi Canullo* *Eitan Mijiritsky* *Silvio Mario Meloni*
